# Nanochannel-based heterometallic {Zn^II^Ho^III^}–organic framework with high catalytic activity for the chemical fixation of CO_2_†

**DOI:** 10.1039/d1ra00590a

**Published:** 2021-03-04

**Authors:** Tao Zhang, Hongtai Chen, Hongxiao Lv, Qiaoling Li, Xiutang Zhang

**Affiliations:** North University of China Taiyuan 030051 People's Republic of China qiaolingl@163.com xiutangzhang@163.com; Taiyuan Institute of Technology Taiyuan 030008 People's Republic of China

## Abstract

The exquisite combination of Zn^II^ and Ho^III^ generated the highly robust [ZnHo(CO_2_)_6_(OH_2_)]-based heterometallic framework of {[ZnHo(TDP)(H_2_O)]·5H_2_O·3DMF}_*n*_ (NUC-30, H_6_TDP = 2,4,6-tri(2′,4′-dicarboxyphenyl)pyridine), which featured outstanding physicochemical properties, including honeycomb nanochannels, high porosity, large specific surface area, the coexistence of highly open Lewis acid–base sites, good thermal and chemical stability, and resistance to most organic solvents. Due to its extremely unsaturated metal tetra-coordinated Zn(ii) ions, hepta-coordinated Ho(iii) and high faveolate void volume (61.3%), the conversion rate of styrene oxide and CO_2_ into cyclic carbonates in the presence of 2 mol% activated NUC-30 and 5 mol% *n*-Bu_4_NBr reached 99% under the mild conditions of 1.0 MPa and 60 °C. Furthermore, the luminescence sensing experiments proved that NUC-30 could be used as a fast, sensitive and highly efficiency sensor for the detection of Fe^3+^ in aqueous solution. Therefore, these results prove that nanoporous MOFs assembled from pyridine-containing polycarboxylate ligands have wide applications, such as catalysis and as luminescent materials.

## Abstract 2

The robust, double-walled, honeycomb material {[ZnHo(TDP)(H_2_O)]·2DMF·4H_2_O}_*n*_ exhibits an excellent catalytic performance in the chemical fixation of CO_2_ and the efficient detection of Fe(iii) ions in aqueous solution.

## Graphical abstract

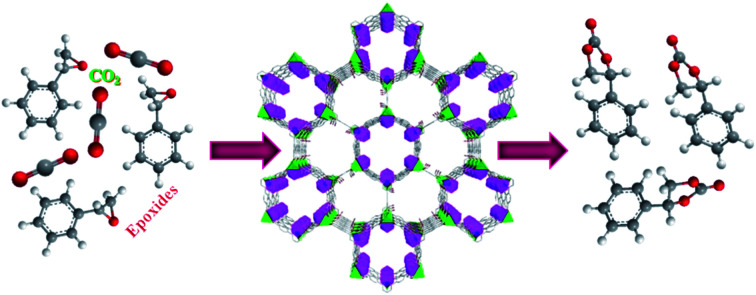

## Introduction

Porous metal–organic frameworks (MOFs), as a booming family of organic–inorganic hybrid materials, have aroused much interest owing to their potential applications as heterogeneous catalysts, gas separation and storage materials, biomedical and chemical sensors, electrochemical microelectrodes, *etc.*^1^ With the development of technology and social advancement, the highly optimized synthesis of targeted MOFs has been adopted and applied stepwise by combining functionalized organic linkers and special metal clusters to realize excellent functional characteristics including inherent porosity, large surface area, and active sites.^2^ Thus far, numerous MOFs have been widely applied as a type of effective separation and catalytic material for the separation and chemical conversion of CO_2_, which has created the serious problem of global warming because of excessive emissions from modern enterprises and motorized tools.^3^ Particularly, the mild reaction conditions of low temperature and pressure utilizing MOF catalysts are incomparable with other hetero- and homogeneous catalysts, for instance, zeolites, inorganic metal salts or oxides, ionic liquids, amine aqueous solutions, and polymerized organics.^4^ Furthermore, MOFs (*e.g.*, MOF-74/CPO-27 series),^5^ as a type of organic–inorganic hybrid material with marvelous structural adaptability, feature the excellent characteristics of high specific surface area and synergistic micropore size, consequently leading to a better unique adsorption performance for CO_2_. In particular, the MOF-74 series of modified M_2_(dobpdc) (dobpdc = 4,4-diphenyl-3,3-dicarboxylate) has been functionalized with diamine to exhibit excellent CO_2_ absorption and selectivity under humid conditions, thus eliminating the water limitation.^6^ For instance, in traditional aqueous amine solutions, the diamine-grafted M_2_(dobpdc) series fully demonstrates the advantage of Lewis acid–base sites, and thus CO_2_ molecules can react with amines to form ammonium carbamate or bicarbonates. Therefore, due to the lower renewable energy cost from solid materials, diamine-appended M_2_(dobpdc) materials with high working ability and energy efficiency are very promising candidates for CO_2_ capture. Moreover, to promote the selective adsorption capacity of CO_2_ in terms of kinetics and thermodynamics, research interest on MOFs at present is concentrated on their performance optimization by designing functional organic ligands to adjust their pore size, increasing open metal sites to improve their adsorption affinity for CO_2_, and improving their thermal and chemical stability (*e.g.*, water resistance).^7^

Among the reported MOFs, Zn^2+^ ions, as one type of d-block metal ions and potential strong Lewis acid, have been paid close attention due to their distinctive characteristics including electronic configuration, binding energy, and charge distribution, which render various coupling affinities to multifarious donor atoms from ligands.^8^ Recently, reported Zn-MOFs based on tetra-coordinated Zn^2+^ ions with solvent-free channels displayed good selective adsorption performances for the guest molecules of CO_2_ and catalyzed the chemical transformation of epoxides with CO_2_ into the corresponding alkyl carbonates. This is because the exposed active Zn^2+^ ions act as a strong Lewis acid to polarize and activate CO_2_ and the ring of ethylene oxide *via* coordination affinity.^9^ Thus, the self-assembly of nanoporous Zn-MOFs with the purpose of enhancing the amount of exposed active tetra- or penta-coordinated Zn^2+^ cations will become a research hotspot, especially for application in the selective separation or/and storage of guest molecules, catalysis for some specific reactions, and optics.^10^ Moreover, in the past decade, the research on the structure and chemical bond theory of Ln-MOFs has constantly increased, leading to a great deal of new research branches, such as catalysts, monomolecular magnets, magnetic refrigeration materials, and fluorescence recognition.^11^ Thus far, for the reported Ln-MOFs, it has been exhibited that the coordination modes of Ln^3+^ can vary in a wide range with the largest coordination number of 12 due to the various orbital hybridization of f^*n*^d^2+*m*^sp^3^ (*n* = 0–3, *m* = 1–3) by the electron orbitals of 6s, 6p, 5d, and 4f, which lead to a series of characteristics corresponding with the intrinsic structures.^12^ Thus, although the Ln^3+^ ions included in the reported Ln-MOFs display octa- and nano-coordinated modes, they can still possess strong Lewis acidity and show strong affinity to small guest molecules with smaller steric resistance, such as CO_2_, SO_2_, N_*X*_O_*Y*_, and H_2_S. Recently, the metal–organic framework of [Eu(BTB)(phen)] reported by Zhao and coworkers,^12*c*^ as the first example of Ln-MOFs for the fixation of CO_2_, displayed excellent catalytic activity for the chemical transformation of epoxides and CO_2_ into carbonates under mild conditions, which further confirmed that the stable octa- and nano-coordinated state of Ln^3+^ ions could be viewed as a strong Lewis acid to polarize and activate approaching guest molecules.^13,14^

In addition, some metal cations are also widely found in nature and living organisms, such as Fe^3+^, which is one of the most crucial metal ions in the Earth's crust and biological systems, playing an important role in oxygen transportation, enzyme catalysis, cell metabolism, and DNA and RNA synthesis. A certain amount of Fe^3+^ is necessary to promote the formation of muscle and hemoglobin. Thus, insufficient or excessive Fe^3+^ can lead to physical diseases such as anemia, cirrhosis, heart failure, diabetes, cancer and decreased immunity. Excessive Fe^3+^ in the body can lead to neurodegenerative diseases, such as Alzheimer's disease and Parkinson's disease. Moreover, Fe^3+^ is a common inorganic pollutant in the aquatic environment, which can cause perpetual damage to human health and living surroundings even at low concentrations. Consequently, studies on the effective detection of Fe^3+^ are particularly important. However, traditional analytical methods, such as ion mobility spectroscopy (IMS), inductively coupled plasma (ICP), X-ray dispersion, atomic absorption spectrometry, are generally expensive and time-consuming.^15^

Considering the above discussion and our recent research, heterometallic MOFs with a high porosity distribution were explored by employing the pyridine-containing ligand of 2,4,6-tri(2′,4′-dicarboxyphenyl)pyridine (H_6_TDP), which possesses the followed characteristics: (i) ample carboxyl groups to bridge multiple metal cations, which is beneficial for the formation of a three-dimensional architecture and (ii) the nitrogen atom of pyridine can serve as a Lewis base site, tending to polarize acidic molecules of CO_2_. Herein, we report a robust, double-walled, nano-channel heterometallic framework of {[ZnHo(TDP)(H_2_O)]·2DMF·4H_2_O}_*n*_ (NUC-30), which is based on the exquisite combination of dinuclear [ZnHo(CO_2_)_6_(H_2_O)] SBUs and TDP^6−^ ligands. Interestingly, the desolvated NUC-30 not only possesses dual nanoscopic channels, high porosity and large specific surface area, but also coexisting Lewis acid–base sites, including 4-coordinated Zn^2+^ ions, 7-coordinated Ho^3+^ ions, uncoordinated carboxyl oxygen atoms, and N_pyridine_ atoms. Thus, as expected, NUC-30 displayed high catalytic activity in the cycloaddition of various epoxides into the corresponding alkyl carbonates. In addition, the luminescence sensing experiments proved that NUC-30 can be used as a fast, sensitive and highly efficiency sensor for the detection of Fe^3+^ in aqueous solution.

## Experimental

### Materials and general methods

2,4,6-Tri(2′,4′-dicarboxyphenyl)pyridine (H_6_TDP) was obtained from Jinan Henghua Sci. & Tec. Co. Ltd and used as received without further refinement. Infrared (IR) spectra were measured using a TENSOR 27 spectrometer in the wavenumber range of 600–4000 cm^−1^. Thermogravimetric analysis (TGA) was carried out using a TG-209F3 thermal analyzer at a heating rate of 10 °C min^−1^ under an Ar stream. The powder X-ray (PXRD) diffraction analysis of the complexes was performed on a Rigaku SmartLab (9 kW) diffractometer with Cu-Kα radiation at room temperature at the scanning speed of 10° min^−1^. The SEM-EDS images of NUC-30 were observed on a JSM-7200F scanning electron microscope (SEM, JEOL) at an acceleration voltage of 10.0 kV. Fluorescence spectra were measured using a Hitachi F-4600 fluorescence spectrophotometer at room temperature. The cryogenic N_2_ adsorption and adsorption–desorption isotherms of CO_2_ at 273 K and 298 K were measured on an ASAP 2020 Plus instrument.

### Preparation of NUC-30

A homogeneous solution of anhydrous zinc chloride (0.014 g, 0.10 mmol), Ho_2_O_3_ (0.019 g, 0.05 mmol), H_6_TDP (0.034 g, 0.06 mmol), 7 mL DMF, 2 mL H_2_O, and 0.2 mL HNO_3_ in a 25 mL autoclave was heated at 110 °C for 4 days and then gradually cooled to room temperature at a rate of 10 °C h^−1^. Colourless crystals of NUC-30 were collected by filtration and washed with DMF/H_2_O (yield: 82% based on H_6_TDP). Anal. calcd for NUC-30 (C_29_H_11_HoNO_13_Zn): C, 42.91%; H, 1.36%; N, 1.72%. Found: C, 42.57%; H, 1.42%; N, 1.74%. IR (KBr pellet, cm^−1^): 3422 (vs), 1604 (vs), 1373 (vs), 1105 (w), 1020 (w), 855 (w), 770 (s), 672 (w).

### X-ray crystallography

The diffraction intensity data for NUC-30 was obtained at 296(2) K using a Bruker Smart-APEX II CCD area detector (Mo-Kα radiation, *λ* = 0.071073 nm) with graphite monochromated radiation. The reflection data was corrected for empirical absorption corrections and Lorentz and polarization effects. The structure was solved by direct methods and refined by full-matrix least-squares with the SHELXL package. All non-hydrogen atoms were refined anisotropically. Hydrogen atoms except those on water molecules were generated geometrically with fixed isotropic thermal parameters and included in the structure factor calculations. The block of SQUEEZE in PLATON was employed to eliminate the highly disordered solvent molecules. The crystallographic data and refinement parameters for NUC-30 are listed in Table S1.† Selected bond lengths and angles for NUC-30 are presented in Table S2.† Further details on the crystal structure investigations can be obtained from the Cambridge Crystallographic Data Centre, with the depository number CCDC-2002682 for NUC-30.

### PXRD and thermal analyses

To detect the phase purity, the samples of NUC-30 and H_6_TDP were characterized *via* X-ray powder diffraction at the scanning speed of 10° min^−1^ in the 2*θ* range of 5–30°. As shown in Fig. S1,† the powder X-ray diffraction pattern of NUC-30 after activation was almost the same as the simulated pattern. The IR spectra of NUC-30 and H_6_TDP were measured in the frequency range of 600–4000 cm^−1^, as shown in Fig. S2.† The SEM-EDS images of NUC-30 illustrated that C, O, N, Zn and Ho were evenly distributed on the crystal surface (Fig. S3†). The thermal stability of NUC-30 was tested by thermogravimetric analysis (TGA) and the result is shown in Fig. S4.† According to the TGA curve, the first weight loss of 8.75% in the temperature range of 25–120 °C is consistent with the associated water molecules. The second weight loss in the range of 120–400 °C is ascribed to the solvent of DMF. The framework remained stable up to 400 °C, indicating that NUC-30 exhibits high thermal stability.

### Catalytic experiment operation

The activated sample of NUC-30 was obtained by immersing the newly synthesized crystals into methanol for one week, during which methanol was replaced three times at ambient temperature. Subsequently, the filtered crystals were dried in a vacuum drying oven at 393 K for one day. The catalytic cycloaddition reactions were performed in a 25 mL stainless clave under the solvent-free conditions of 10 atm CO_2_ gas and 2 mmol% heterogeneous catalyst NUC-30. The qualitative detection during the reactive process was monitored by gas chromatography and the transformed product was determined by ^1^H NMR spectroscopy. At the end of the catalytic reaction, the heterogeneous catalyst NUC-30 was retrieved through simple centrifugation separation after each reaction was complete, and then cleaned with strong polar solvent of DMF and volatile solvent of acetone in sequence.

## Result and discussion

### Description of the crystal structure

The single-crystal XRD analysis exhibited that NUC-30 crystallizes in the trigonal system with the *R*3̄*m* space group and exhibits a honeycomb anionic structure with dual-channels, which is built on the exquisite combination of paddlewheel [ZnHo(CO_2_)_6_(H_2_O)] SBUs and bifunctional ligands of TDP^6−^. Although both types of alternately arranged nano-channels (I and II) in NUC-30 shaped by six rows of [ZnHo(CO_2_)_6_(H_2_O)] SBUs have an equal amount of exposed active metal sites, they could be discriminated from their different functionalized inner surfaces with free carboxyl oxygen atoms (C=O) and coordinated aqueous molecules (H_2_O). It is worth mentioning that NUC-30 features outstanding physicochemical properties including honeycomb nanochannels, high porosity, large specific surface area, coexisting highly open Lewis acid–base sites, good thermal and chemical stability, and resistance to most organic solvents. In addition, the activated NUC-30 with void volume (61.3%) consists of extremely unsaturated metal ions including tetra-coordinated Zn(ii) and hepta-coordinated Ho(iii), which render the host framework a promising heterogeneous catalyst for the chemical fixation of CO_2_.

**Fig. 1:**
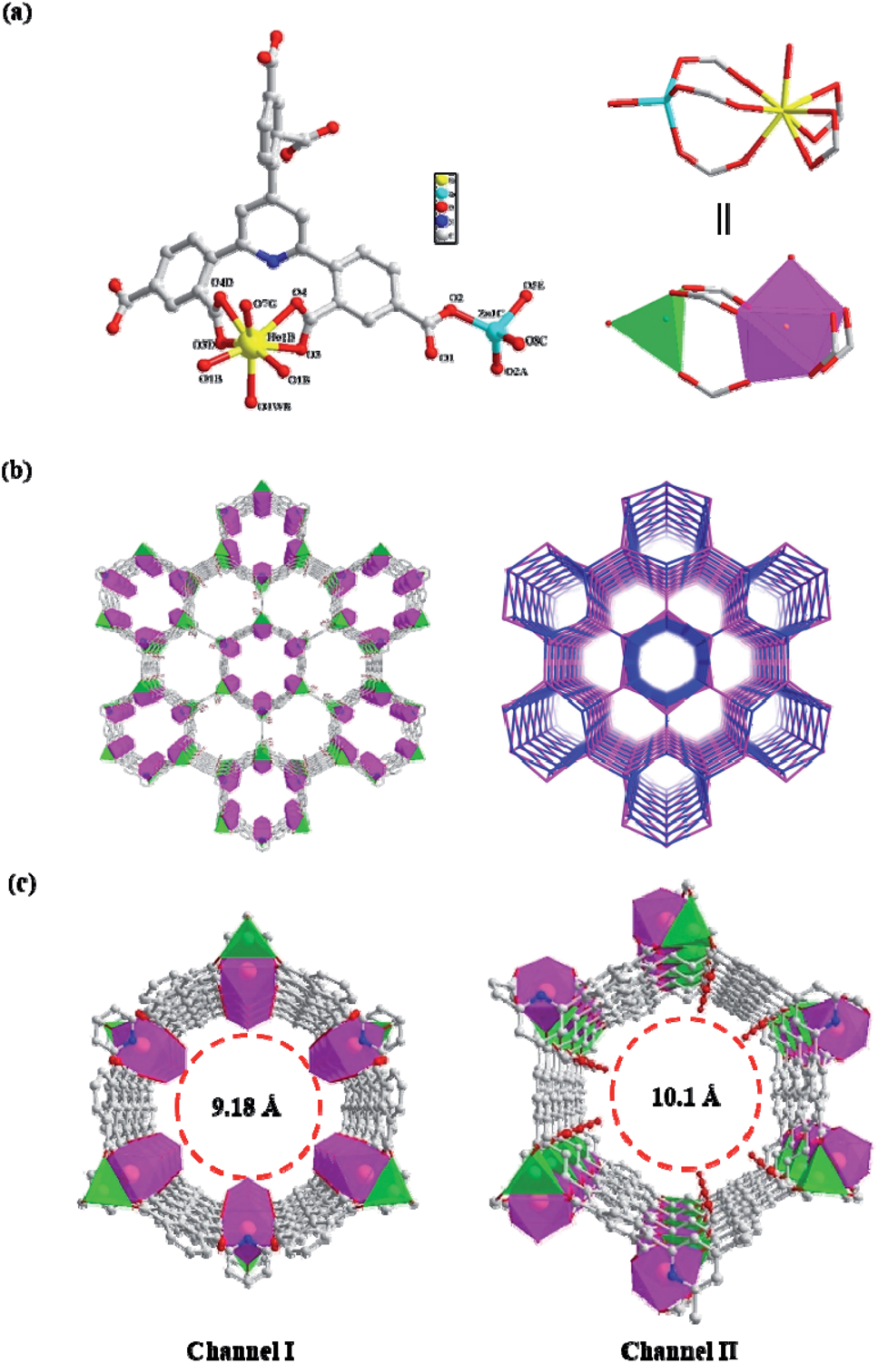
(a) Structural building block of the TDP^6−^ ligand and [HoZn(CO_2_)_6_(H_2_O)] (C, gray; O, red; Zn, cyan; Ho, yellow; N, blue; and H atoms are omitted for clarity). (b) 3D dual-channel framework of NUC-30 and its topology. (c) View of the 3D channel structure of NUC-30 along the *c*-axis.

Specifically, the asymmetric unit of NUC-30 contains half a crystallographically unique Zn(ii) ion, half an Ho(iii) ion, and half a TDP^6−^ ligand. Firstly, the Ho(1) ion is chelated by two α-carboxylic groups with the coordination mode of μ_2_-η^1^:η^1^ located on the 2-position of the phenyl rings, which are arranged on the 2- and 6-positions of the pyridine in TDP^6−^. Afterwards, the Ho(1) ion is further bridged to the Zn(1) ion to form one paddle-wheel dinuclear SBU of [Zn(CO_2_)_6_] with the separate Ho(1)⋯Zn(1) distance of 4.04(9) Å by three other γ-carboxyl groups, two of which are provided by two benzene rings on the 2-position of pyridine and one on the 4-position of pyridine (Fig. 1a). In addition, the Zn(1) ion is coordinated by one 2-position carboxyl group from another TDP^6−^ ligand. Thus, the formation of one [ZnHo(CO_2_)_6_(H_2_O)] SBU is facilitated by five TDP^6−^ ligands, each of which simultaneously connects five [ZnHo(CO_2_)_6_(H_2_O)] SBUs to generate one three-dimensional structure with honeycomb-like nanochannels (Fig. 1b). Although the Ho(1) ion is linked by seven carboxyl oxygen atoms and one aqueous molecule to constitute a “twisted double hat tri-prism” geometry (Fig. S5a†), it is worth mentioning that the associated water molecules can be easily removed under the activation conditions. In addition, the Zn(1) ion is linked by four carboxyl oxygen atoms from four different TDP^6−^ ligands to form a tetrahedron (Fig. S5b†) with the Zn(1)–O bond length in the range of 1.918(2)–1.963(2) Å. Even more exciting is that the tetrahedral Zn(1) in a tetrahedral coordination geometry is a natural Lewis acid site.

It is worth noting that each six rows of [ZnHo(CO_2_)_6_(H_2_O)] SBUs are alternately bridged by TDP^6−^ ligands to form two types of edge-sharing nanochannels, namely circular and hexagonal ones. The circular nanochannels (I) with a pore diameter of 9.18 Å are characterized by the associated water molecules on the Ho^3+^ cations, pointing vertically to the center of the nanochannel. However, the hexagonal nanochannels (II) with an aperture of 10.1 Å show that the free carboxyl oxygen atoms (C=O) reside on the inner walls, as shown in Fig. 1c. Therefore, channel I and channel II have completely different functionalized inner surfaces, except for the C–H organic panels and similar exposed metal parts on the walls.

Finally, for clarity, in the structure of NUC-30, the host framework was simplified as an fng-type framework with the Schläfli symbol of {4^6^·6^4^} as analyzed by TOPOS40. In the topology, the binuclear [HoZn(CO_2_)_6_(H_2_O)] SBUs and trigeminal TDP^6−^ ligand are simplified as five-connected nodes, as shown in Fig. 1b.

### Water resistance of NUC-30

Due to the obvious advantages of MOFs in gas-phase and liquid-phase adsorption fields, there is a lot of emphasis on their stability in the chemical environment. However, the low water resistance of most MOFs limits their application in the aqueous phase.^15^ Therefore, the water resistance of NUC-30 in different aquatic systems was analyzed. As shown in Fig. S6,† the PXRD spectra demonstrated that no structural collapse occurred after NUC-30 was soaked for 10 days at normal temperature, and even after soaking in boiling water for 72 h, which reflects its strong water resistance. This may be due to the change in the internal environment of its nanochannel by heterometallic doping, resulting in the strong hydrophobicity of the inner surface. The method of controlling the stability of MOFs by heterometallic doping also provides a new idea for improving the properties of porous metal–organic frameworks.

### Gas adsorption studies

To explore the permanent porosity of NUC-30, crystal samples were soaked in methanol and dichloromethane separately three times a week, the solvent was changed every 24 h, and then dried under vacuum at 120 °C for 12 h. The PXRD pattern of the activated NUC-30 was consistent with the results of the X-ray single crystal diffraction data simulation, as shown in Fig. S7.† The adsorption capacity for N_2_ at 77 K displayed that the total pore volume and calculated BET surface area of the activated NUC-30 were 0.38 cm^3^ g^−1^ and 701 m^2^ g^−1^, respectively. The adsorption–desorption isotherm of N_2_ displayed a typical type-I adsorption configuration, and the hole distribution was in the range of 0.9–1.3 nm, as shown in Fig. S8.†

**Fig. 2:**
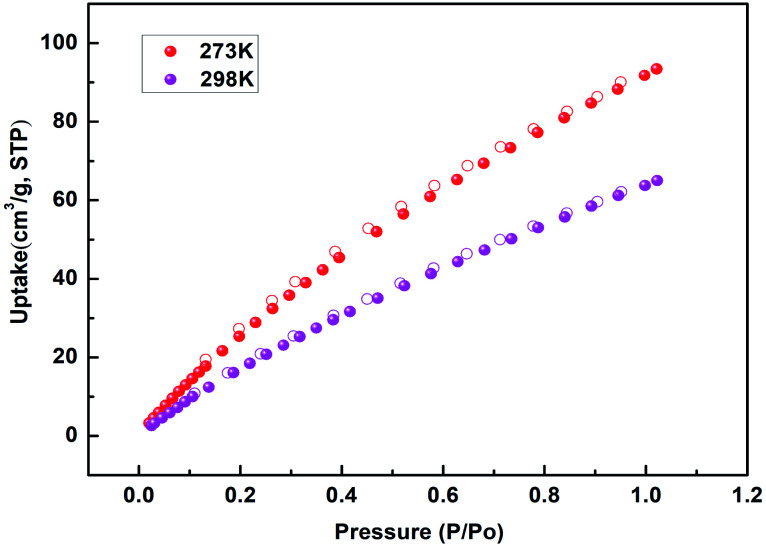
CO_2_ sorption isotherms of NUC-30 at 273 and 298 K.

Due to the inherent structural advantages of the tubular nanochannels and the exposed high-density active metal sites of NUC-30, it has the potential to adsorb target molecules with quadrupole moments, and thus the adsorption of CO_2_ was explored at 273 K and 298 K.^16,17^ As shown in Fig. 2, the maximum absorption of CO_2_ at 273 K and 298 K was 93.44 cm^3^ g^−1^ and 64.99 cm^3^ g^−1^, respectively, which are higher than that in the literature, such as for NUC-5, MOF-5, and Uio-66.^18^ Simultaneously, incomplete adsorption isotherms and CO_2_ desorption lead to a moderate hysteresis loop, which confirms the strong interaction between the host and guest. Furthermore, in quantify the binding force between the CO_2_ molecules and the framework of NUC-30, *Q*_st_ was calculated using the virial method with the resulting value at zero coverage being 25.8 kJ mol^−1^, which implies that the adsorbed CO_2_ molecules can be easily released, allowing the host framework of NUC-30 to be regenerated, as shown in Fig. S9.†

### Catalytic cycloaddition of CO_2_ and epoxides

On account of the abovementioned characteristics including highly exposed metal cations, highly partitioned functionalized nanochannels, large specific surface area, and good trapping performance of CO_2_, the catalytic efficiency of NUC-30 was tested for the chemical transformation of epoxides with CO_2_ under comparatively mild conditions into cyclic carbonates, which is viewed as a bifunctional reaction for synergistically solving the energy crisis and reducing the amount of greenhouse gases.^19^ As illustrated in Table 1, 1,2-epoxypropane was selected as a model to explore the effects of different reaction conditions on the conversion of propylene oxide from CO_2_ to propylene carbonate, and the obtained products were determined by ^1^H NMR and GC-MS spectroscopy. Throughout the experimental process, the amount of NUC-30 was set at 1.0 mol%. Entry 1 in Table 1 shows that the yield was 13% only in the presence of only solvation-free NUC-30 at room temperature, while the co-catalyst Bu_4_NBr under similar reaction conditions had a yield of 6% (entry 2). In contrast, as can be seen from entry 3, when 1.0 mol% NUC-30 and 2.5 mol% Bu_4_NBr were used simultaneously, the yield reached 49%, which proved that NUC-30 and Bu_4_NBr possessed completely different catalytic efficiency for the second-order reaction of cycloaddition from epoxides with CO_2_. In addition, with an increase in temperature, the yield increased correspondingly, and the final yield reached 99% after 24 h at 60 °C (entry 6). However, when the amount of Bu_4_NBr was doubled and NUC-30 remained unchanged, the yield increased to 98% within 8 h at 60 °C (entry 10). Furthermore, as shown in entries 1 and 2, the unsatisfactory results obtained by employing single NUC-30 or *n*-Bu_4_NBr demonstrated that the active open metal sites of NUC-30 and anions of Br^−^ played different roles during the cycloaddition reaction of CO_2_ and epoxides. Therefore, the optimal reaction conditions used for subsequent experiments were quantified as 1.0 mol% NUC-30 catalyst, 5 mol% *n*-Bu_4_NBr co-catalyst, and 1.0 MPa CO_2_ at 60 °C for 8 h. Compared with the recently reported MOF-based catalysts,^20^NUC-30 showed a superior catalytic performance in terms of reaction conditions (Table S4†), which should be attributed to its intrinsic characteristics of active metal sites (Zn^II^ and Ho^III^), uncoordinated pyridine and carboxyl oxygen atoms, and unimpeded void space. Furthermore, besides the synergistic catalytic effect of NUC-30 and *n*-Bu_4_NBr, the reaction temperature plays a key role during the cycloaddition process.

**Table 1:** Cycloaddition reaction of CO_2_ with styrene oxide under various conditions^a^

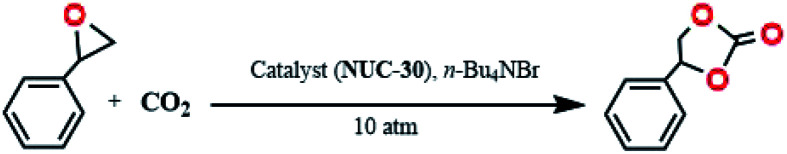					
Entry	NUC-30 (mol%)	*n*-Bu_4_NBr (mol%)	*T* (°C)	*t* (h)	Yield^b^ (%)
1	1.0	0	25	24	13
2	0	2.5	25	24	6
3	1.0	2.5	25	24	49
4	1.0	2.5	40	24	72
5	1.0	2.5	50	24	85
6	1.0	2.5	60	24	99
7	1.0	5.0	60	2	41
8	1.0	5.0	60	4	65
9	1.0	5.0	60	6	88
10	1.0	5.0	60	8	98

a Reaction conditions: 20 mmol propylene oxide, solvent free, CO_2_ (10 atm).

b Checked by ^1^H NMR and GC-MS spectroscopy with *n*-dodecane as the internal standard.

Under the established optimal reaction conditions including 20 mmol epoxide, 1.0 mol% NUC-30, 5 mol% *n*-Bu_4_NBr, and 10 atm CO_2_ at 60 °C for 8 h, a series of epoxide derivatives with typical substitutes was used to check the catalytic universality of NUC-30. As demonstrated in Table 2, the activated NUC-30 exhibited significant catalytic activity for all the chosen substrates with yields higher than 96% under the set reaction conditions, except for styrene oxide with the relatively high boiling point of 194 °C, which may have limited its activity and rendered the final yield lower at the low temperature of 60 °C. The comparison of entries of 1–4 exhibited that epoxides with single electron-withdrawing groups are more likely to undergo the cycloaddition reaction, which should be attributed to the fact that the electron absorption effect can effectively reduce the electron density of ethylene oxide. Furthermore, by comparing entries 1–4 and 5, it is clear that steric hindrance has a great influence on the cycloaddition reaction, which is consistent with the existing similar reactions catalyzed by porous MOFs.^21,22^ According to our hypothesis, the reason for this is that the larger substituents on the epoxides not only affect the convenience of their access to the nanocages, but also reduce the opportunity of interaction with the active sites. Moreover, compared to the reported porous MOF-based catalysts of MOF-205(S),^23*a*^MMCF-2,^23*b*^ and Hf-Nu-1000,^23*c*^NUC-30 exhibits superior catalytic efficiency, which should be ascribed to the contribution of the [ZnHo(CO_2_)_6_(H_2_O)] units on the surface of its channels based on the clarified reaction mechanism. During the reaction process, the principle of catalysis is to reduce the reaction energy level and accelerate the reaction process by polarizing the reactants of epoxides and CO_2_.

**Table 2:** Cycloaddition reaction of CO_2_ and various epoxides with NUC-30 as the catalyst^a^

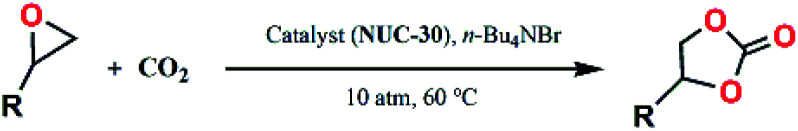				
Entry	Epoxide	Product	Yield^a^^,^^b^ (%)	Selectivity (%)
1	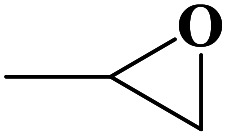	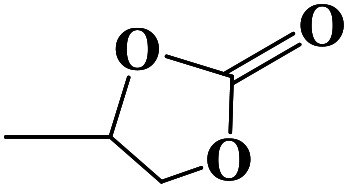	99	>99
2	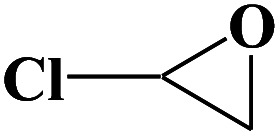	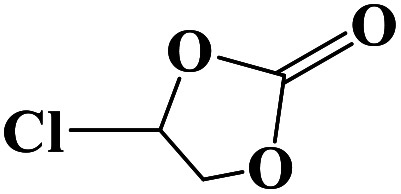	99	>99
3	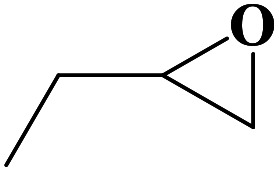	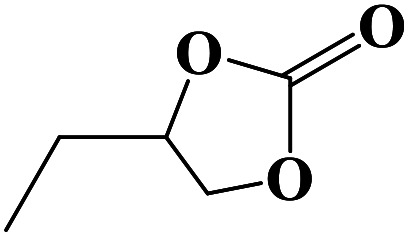	98	>99
4	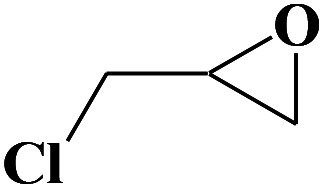	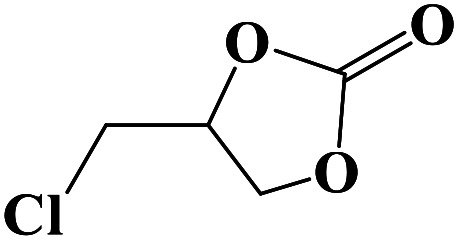	96	>99
5	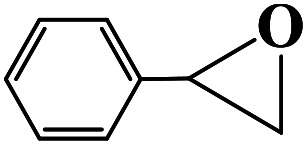	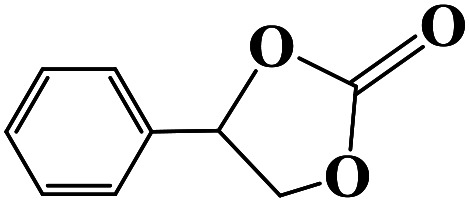	92	>99

a Reaction conditions: substrate (20 mmol), *n*-Bu_4_NBr (5 mol%), NUC-30 catalyst (1.0 mol% based on the Zn metal center), CO_2_ (10 atm), 60 °C, and 12 h.

b Determined by GC/MS with *n*-dodecane as the internal standard.

The post-treatment including recovery and cyclic utilization for a proposed catalyst is one of the most important factors for evaluating its practical application besides excellent catalytic performance. Therefore, the ten-fold amplification reaction based on the optical conditions with 1,2-epoxypropane as the substrate by employing 500 mg NUC-30 was repeated 5 times, during which NUC-30 was recycled by filtration after each cycle and directly reused for the next cycle. When the fifth cycle was completed, the recovered sample of NUC-30 was rinsed with dichloromethane, and subsequently vacuum dried, leaving a weight of 482 mg with the loss of 3.6%. The ICP analysis of the reaction solution showed that there was only a trace amount of Zn(ii) (≈0.027%) and Ho(iii) (≈0.018%) ions leached from the host framework. Notably, the catalytic effect of NUC-30 was almost identical during the five cycles, which was confirmed by the transformation yield, as shown in Fig. S10.† Simultaneously, the PXRD pattern of the recovered NUC-30 sample after five experimental runs was basically consistent with the original pattern, indicating that the structural integrity of NUC-30 was still intact (Fig. S11†). As shown in Fig. S12,† the catalytic reaction was close to inactive after the catalyst was separated from the reaction system for a period of time, which confirmed the catalytic activity of NUC-30.

**Fig. 3:**
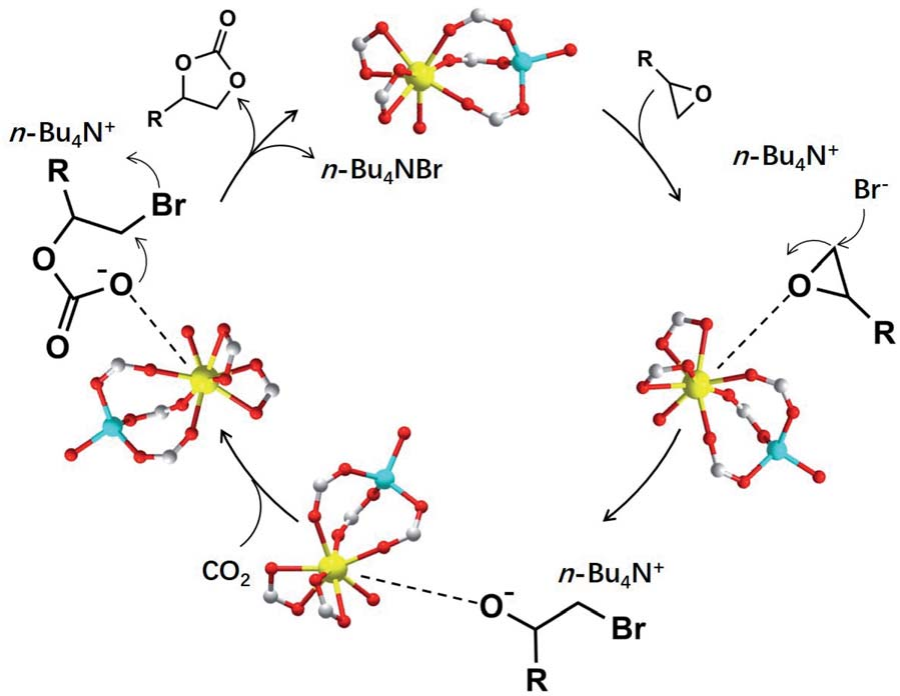
Catalytic mechanism for the cycloaddition of epoxides with CO_2_.

Given the clarified structure of NUC-30 and recently published studies,^6,23^ the speculated catalytic mechanism is shown in Fig. 3. Primarily, the oxygen atom of the selected epoxide interacts with the open zinc sites on the inner wall of the nanotubular channel to decrease the original structural stability of the epoxide ring. Consequently, the bromide atom of TBAB poses nucleophilic attack on the less hindered carbon atom of the activated epoxide to generate one intermediate alkylcarbonate anion, which tends to launch an attack to the neighbouring polarized CO_2_ molecule caught by the Lewis-acid metal sites in the nanochannel. Hence, it can be inferred that the whole process of cycloaddition can be boosted by coordination *via* the weak Lewis acidic–basic interactions involved in the porous framework.

### Luminescence characteristic and sensing property

MOFs with d^10^ transition metal ions as constituents have been reported to be potential fluorescent materials in the field of optics. Consequently, the solid-state luminescence properties of the pure H_6_TDP ligand and NUC-30 at room temperature were studied, as shown in Fig. S13.† The maximum emission peaks of H_6_TDP and NUC-30 appeared at ∼400 nm and ∼410 nm, respectively. Compared with the H_6_TDP ligand, the maximum emission of NUC-30 has a weak red shift. Furthermore, due to their d^10^ electronic configuration, it is difficult to oxidize or reduce the Zn(ii) and Ho(iii) ions. Moreover, compared with the free H_6_TDP, the emission of NUC-30 was observed to be red-shifted, which is mainly due to the coordination effect between the ligand and metal ions. In addition, the excellent water-resistance of NUC-30 makes it a potential luminescence probe to detect different metal ions.

### Determination of Fe^3+^ cation

**Fig. 4:**
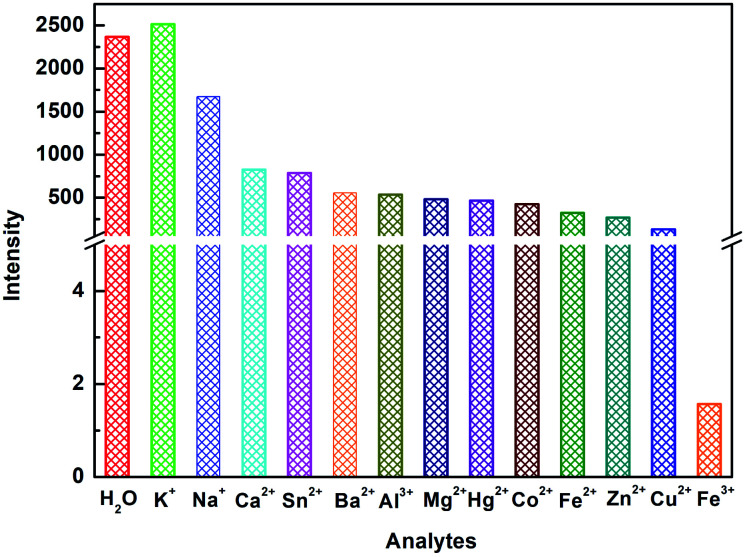
Luminescence intensity of NUC-30 at 397 nm in various cations.

**Fig. 5:**
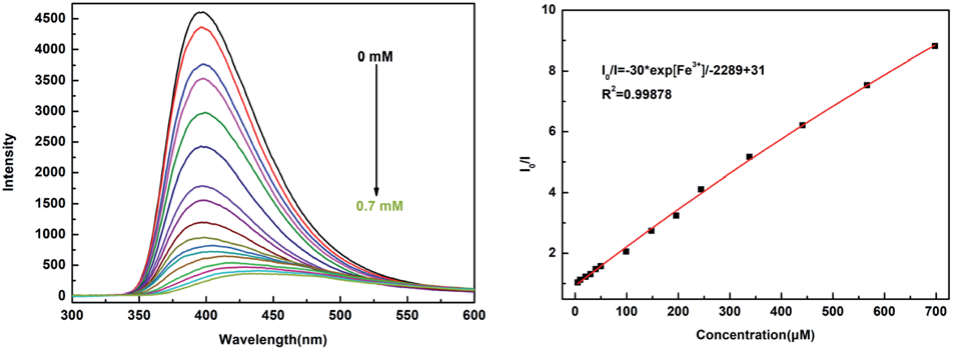
(a) Luminescence spectra of the NUC-30@Fe^3+^ suspensions with the Fe^3+^ concentration varying from 0 to 0.75 mM (excited at 397 nm). (b) Plot of relative intensity *vs.* Fe^3+^ concentration.

The investigation of the sensing ability of NUC-30 for various cations was studied by adding 2 mg samples in 2 mL of 0.01 M aqueous solutions of twelve cations, M(Cl)_*x*_ (M = Na^+^, Zn^2+^, Co^2+^, Hg^2+^, K^+^, Ca^2+^, Ba^2+^, Cu^2+^, Mg^2+^, Al^3+^, Fe^3+^, Fe^2+^, and Sn^2+^) in water at room temperature. As seen in Fig. 4, the luminescent intensity of the M^*n*+^@CP suspensions varied with the different types of metal cations. The results exhibited that the Fe^3+^ cation has the best luminous quenching effects on NUC-30. The relationship between the concentration of Fe^3+^ cations and the luminescent intensity of the NUC-30 suspension was studied synchronously. As seen in Fig. 5, the fluorescent intensity of NUC-30 displayed an obvious quenching effect with the addition of Fe^3+^ solution. When the concentration of Fe^3+^ reached 0.7 mM, the quenching rate of NUC-30 increased to 97% (Fig. 5a). The non-linear Stern–Volmer curve for NUC-30@Fe^3+^ in aqueous system is consistent with the exponential equation of *I*_0_/*I* = −30 × exp[Fe^3+^]/−2289 − 31 for NUC-30 (Fig. 5b). In addition, as shown in Fig. S14,†NUC-30 had a high *K*_SV_ value of 2.413 × 10^4^ M^−1^ for Fe^3+^ ions by fitting the experimental database in the low concentration range. Furthermore, according to the *K*_SV_ value and the standard deviation (*δ*), the detection minimum limit of NUC-30 for Fe^3+^ cations was determined to be 0.17 μM.

In addition, the infrared spectrum (IR) and PXRD patterns of the samples after the fluorescent experiment were consistent with the original samples, which shows that the structure of NUC-30 did not change (Fig. S15 and S16,† respectively). In addition, according to the structural characteristics of NUC-30 and the related studies on fluorescence recognition by MOFs,^24^ the possible luminescence quenching mechanism of NUC-30 toward Fe^3+^ cations was further investigated. The ICP results showed that no Ho^3+^ ions were detected in the NUC-30@Ho^3+^ suspension after the luminescence experiments, indicating that the fluorescence quenching was not caused by ion exchange (Table S5†) and may be caused by the Fe^3+^ cations involved in the competitive absorption of the excitation light between them and the CPs, and the energy resonance transfer from the framework of NUC-30 to the Fe^3+^ cations.

## Conclusions

By utilizing the ligand-directed synthetic strategy, the solvothermal self-assembly of Zn^2+^ and Ho^3+^ ions with the aid of the pre-designed H_6_TDP generated the robust, double-walled, honeycomb material NUC-30, which exhibited excellent catalytic performances for the chemical transformation of various epoxides into the corresponding carbonates under comparatively mild conditions of 10 atm CO_2_ flow and 60 °C. Meanwhile, the water-resistant framework of NUC-30 could selectively and sensitively discriminate Fe^3+^ ions in aqueous solution. In fact, NUC-30 represents a class of emerging heterometallic nanoporous materials based on heterometallic binuclear SBUs. These materials not only feature the characteristics of dual tubular nanochannels, high porosity and specific surface area, but also possess tetra-coordinated transition metal ions and octa-coordinated rare earth ions. Although the ions of Zn^2+^ and Ho^3+^ were selected to construct the targeted model for the catalytic study on the chemical fixation of CO_2_ by our group, other combinations of transition metal ions and octa-coordinated rare earth ions with the aid of H_6_TDP can also be assembled for other specific functional studies in the future, such as optics and magnetism.

## Conflicts of interest

The authors have no conflicts of interest to declare.

## Supplementary Material

RA-011-D1RA00590A-s001

RA-011-D1RA00590A-s002
