# Supplementary material for: Nanochannel-based heterometallic {ZnIIHoIII}–organic framework with high catalytic activity for the chemical fixation of CO2
Source: RSC Adv. 2021 Mar 4;11(16):9731–9. doi: 10.1039/d1ra00590a (PMC8695428; doi:10.1039/d1ra00590a)
Supplement: RA-011-D1RA00590A-s001 [file RA-011-D1RA00590A-s001.pdf]

Electronic Supplementary Material (ESI) for RSC Advances.
This journal is © The Royal Society of Chemistry 2021

*Supporting Information*

# Nanochannel-Based Heterometallic {$Zn^{II}Ho^{III}$}-Organic Framework with Highly Catalytic Activity on Chemical Fixation of $CO_2$

Tao Zhang,[a,b] Hongtai Chen,[a] Hongxiao Lv,[a] Qiaoling Li,[a, *] and Xiutang Zhang[a, *]

*North University of China, Taiyuan 030051, People's Republic of China; *E-mail: qiaolingl@163.com, xiutangzhang@163.com.*
*Taiyuan Institute of Technology, Taiyuan 030008, People's Republic of China.*

# Contents

**X-ray crystallography**

**Table S1. Crystallographic data and refinement parameters of NUC-30.**

**Table S2. Selected bond lengths and angles of NUC-30.**

**Table S3. The molecular size of various substituted epoxides.**

**Table S4. Comparison of the catalytic performance of NUC-30 catalyst with selected previously reported MOFs.**

**Table S5. The ICP measurement results.**

**Figure S1. PXRD pattern of activated NUC-30 and simulated.**

**Figure S2. IR spectrum of as-synthesized NUC-30.**

**Figure S3. SEM-EDS images of NUC-30 (C: Red；N: Yellow; O: Cyan; Zn: Purple; Ho: Green).**

**Figure S4. TGA curve of as-synthesized sample of NUC-30.**

**Figure S5. Ho-o polyhedral structure(a), Zn-o tetrahedral structure(b).**

**Figure S6. PXRD patterns of NUC-30 under water treatment.**

**Isosteric heat calculation.**

**Figure S7. PXRD pattern of activated NUC-30 and simulated**

**Figure S8. $N_2$ absorption and desorption isotherms of NUC-30 at 77 K (Insert: the pore size distribution).**

**Figure S9. $CO_2$ adsorption heat calculated by the virial equation of NUC-30.**

**Yield calculation based on the GC-MS analysis.**

**Figure S10. The recycled cycloaddition reaction of $CO_2$ with styrene oxide.**

**Figure S11. The PXRD pattern of NUC-30 after recycled cycloaddition reaction.**

**Figure S12. Evidence of heterogeneous nature of NUC-30 in the coupling of chloromethyloxiran with $CO_2$.**

**Figure S13. The fluorescent emission curves of NUC-30 (black) and ligand $H_6$TDP (red).**

**Figure S14.The fitting experimental data with low concentration range of $Fe^{3+}$.**

**Figure S15. IR spectrum of NUC-30 under fluorescence quenching of different ions.**

**Figure S16. PXRD patterns of NUC-30 under fluorescence quenching of different ions.**

**X-ray crystallography.**

Single-crystal diffractometry for **NUC-30** was conducted on a Bruker Smart Apex CCD diffractometer of Bruker D8 VENTURE PHOTON II by employing graphite-monochromated Mo-Kα radiation (λ = 0.071073 nm) at 296(2) K and then corrected for Lorentz and polarization effects along with the multi-scan absorption by an correction SADABS program. Therefore, crystal structure was offered by direct methods and refined by full-matrix least-squares with the SHELXL (Sheldrick, 2015) package. Hydrogen atoms except those on water molecules were generated geometrically with fixed isotropic thermal parameters, and included in the structure factor calculations. The solvent content of **NUC-30** was determined with the help of the thermogravimetric analysis (Figure S2). Crystallographic data and refinement parameter were listed in Table S1. Selected bond lengths and angles were concluded in Table S2. Further details on the crystal structure investigations may be obtained from the Cambridge Crystallographic Data Centre, with the depository number CCDC-2036304 for **NUC-30**.

**Table S1. Crystallographic data and refinement parameters of NUC-30.**

| Complex | **NUC-30** |
|---|---|
| Formula | $C_{29}H_{11}HoNO_{13}Zn$ |
| Mr | 811.71 |
| Crystal system | Trigonal |
| Space group | R-3m |
| a (Å) | 47.916 (16) |
| b (Å) | 47.916 (16) |
| c (Å) | 13.299 (4) |
| α (°) | 90 |
| β (°) | 90 |
| γ (°) | 120 |
| V(Å$^3$) | 26443.9(2) |
| Z | 18 |
| Dcalcd(g·cm$^{-3}$) | 0.917 |
| μ(mm$^{-1}$) | 3.249 |
| GOF | 1.028 |
| $R_1$ [I＞2σ(I)]a | 0.0460 |
| $wR_2$ [I＞2σ(I)]b | 0.1573 |
| $R_1^a$(all data) | 0.0479 |
| $wR_2^b$ (all data) | 0.1592 |
| $R_{int}$ | 0.0289 |

$^a R_1 = \sum \left| |F_o| - |F_c| \right| / \sum |F_o|$ . $^b wR_2 = |\sum w(|F_o|^2 - |F_c|^2)| / \sum |w(F_o{}^2)^2|^{1/2}$

**Table S2. Selected bond lengths and angles of NUC-30.**

| **NUC-30** | | | | | |
|---|---|---|---|---|---|
| Ho(1)-O(1) | 2.243(2) | O(2)-C(1) | 1.254(4) | C(15)-C(19) | 1.508(5) |
| Ho(1)-O(1)#1 | 2.243(2) | O(1)-C(1) | 1.249(4) | C(15)-C(14) | 1.305(6) |
| Ho(1)-O(7)#3 | 2.286(3) | O(7)-C(19) | 1.224(5) | C(15)-C(16) | 1.334(6) |
| Ho(1)-O(4)#2 | 2.369(3) | O(4)-C(8) | 1.239(4) | C(11)-C(12) | 1.481(5) |
| Ho(1)-O(4)#7 | 2.369(3) | N(1)-C(9) | 1.372(4) | C(3)-C(2) | 1.393(4) |
| Ho(1)-O(3)#2 | 2.436(3) | N(1)-C(9)#4 | 1.372(4) | C(2)-C(7) | 1.394(4) |
| Ho(1)-O(3) #7 | 2.436(3) | O(3)-C(8) | 1.237(4) | C(12)-C(13) | 1.338(6) |
| Ho(1)-OW | 2.346(4) | O(8)-C(19) | 1.251(5) | C(12)-C(17) | 1.331(7) |
| Ho(1)-C(8)#2 | 2.762(3) | C(9)-C(10) | 1.368(4) | C(6)-C(7) | 1.374(6) |
| Ho(1)-C(8)#7 | 2.762(3) | C(9)-C(5) | 1.476(5) | C(14)-C(13) | 1.376(7) |
| Zn(1)-O(5)#5 | 1.933(3) | C(10)-C(11) | 1.363(4) | C(13)-C(18) | 1.658(7) |
| Zn(1)-O(2) | 1.964(3) | C(4)-C(5) | 1.407(4) | C(13)-C(18)#4 | 1.658(7) |
| Zn(1)-O(2)#1 | 1.963(3) | C(4)-C(3) | 1.381(5) | C(16)-C(17) | 1.368(8) |
| Zn(1)-O(8) #3 | 1.918(3) | C(1)-C(2) | 1.486(5) | C(18)-C(18)#4 | 1.593(17) |
| O(5)-C(18) | 1.239(7) | C(5)-C(6) | 1.387(5) | C(18)-O(6) | 1.198(9) |
| O(5)-C(18)#2 | 1.239(7) | | | | |
| Symmetry transformations used to generate equivalent atoms: #1 -x+y, +y, +z #2 -1/3+y, 1/3+x, 1/3-z; #3 1/3-x+y, 5/3-x, -1/3+z; #4 +x, 1+x-y, z; #5 -1/3+y, 1/3+x, 4/3-z; #6 4/3-x, 2/3-x+y, 2/3-z; #7 2/3+x-y, 1/3+x, 1/3-z. | | | | | |

**Table S3. The molecular size of various substituted epoxides.**

| Entry | Epoxides | Molecular size[a] (Å) |
|---|---|---|
| 1 | 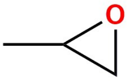 | 6.145*4.454*5.035 |
| 2 | 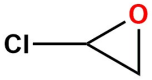 | 6.121*4.397*5.046 |
| 3 | 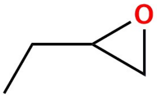 | 7.144*5.296*5.084 |
| 4 | 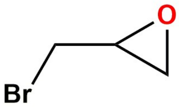 | 7.202*5.611*5.089 |
| 5 | 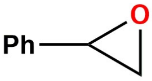 | 9.323*6.898*4.646 |
| 6 | 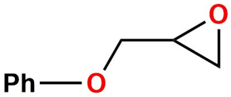 | 11.208*6.765*4.823 |

[a] van der Waals radii were determined by Bondi.

**Table S4. Comparison of the catalytic performance of NUC-30 catalyst with selected previously reported MOFs.**

| MOF | Catalyst (mol %) | Temperature | Pressure (MPa) | Time | Yield (%) | Ref. |
|---|---|---|---|---|---|---|
| **MOF-205(M)** | 2.5 | RT | 0.4 | 4 | 80 | S1 |
| **NH2-MIL-101(Al)** | 0.17 | 120 | 1.8 | 6 | 95 | S2 |
| **MMCF-2** | 0.13 | RT | 0.1 | 48 | 95 | S3 |
| **Cr-MIL-101** | 1.2 | RT | 0.8 | 24 | 82 | S4 |
| **UiO-67** | 1.5 | 90 | 0.1 | 12 | 95 | S5 |
| **MOF-893** | 0.32 | 80 | 0.1 | 23 | 88 | S6 |
| **M-MOF-184** | 1.2 | 80 | 0.1 | 6 | 82 | S7 |
| **NUC-30** | 1.0 | 60 | 1.0 | 8 | 98 | This work |

**Table S5. The ICP measurement results.**

| Fluorescence experiment of **NUC-30** | Initial | After sensing measurement |
|---|---|---|
| The concentration of $Fe^{3+}$ ($mol \cdot L^{-1}$ ) | $1.0\times10^{-2}$ | $9.97\times10^{-3}$ |

ICP measurements:

After the fluorescence experiment, the suspension was filtered. And filter cake (**NUC-30**) was washed several times with fresh solvent. Then, ICP measurements were performed on the filtrate (solution containing $Fe^{3+}$ ions), the results were listed in Table S5.

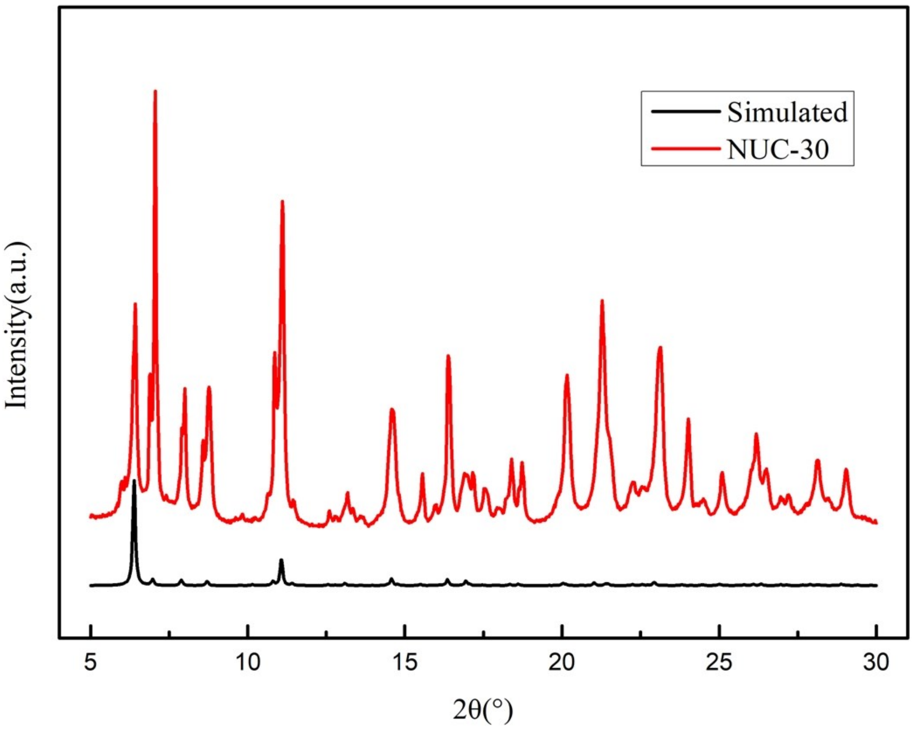


**Figure S1. PXRD pattern of NUC-30 and simulated**

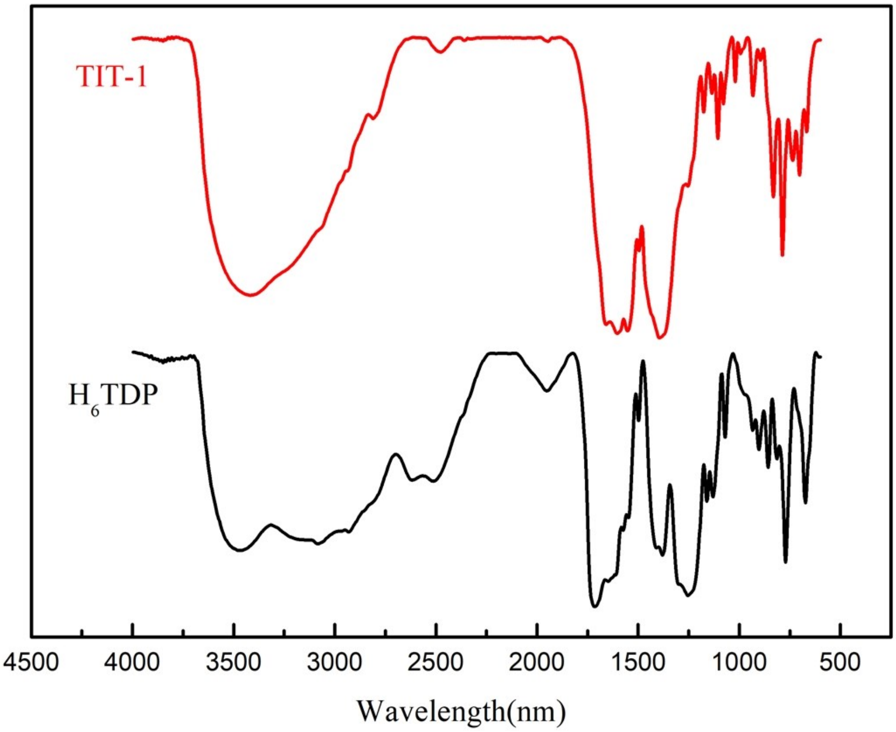

**Figure S2. IR spectrum of as-synthesized NUC-30.**

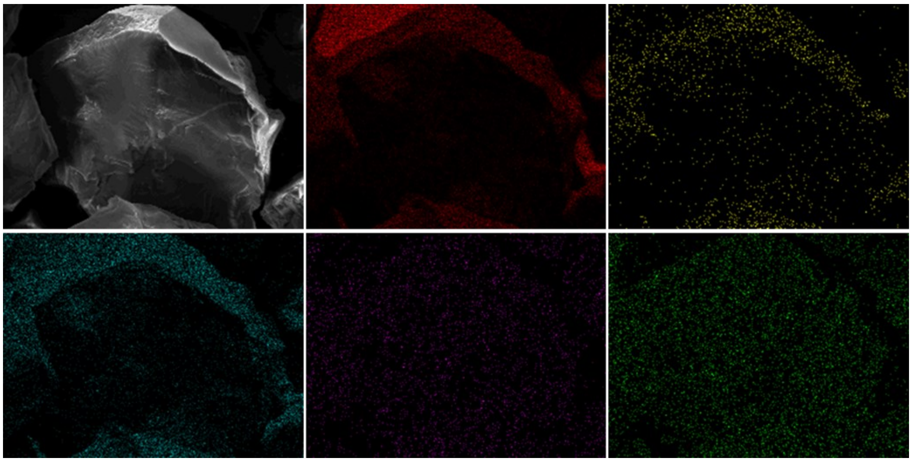

**Figure S3. SEM-EDS images of NUC-30 (C: Red；N: Yellow; O: Cyan; Zn: Purple; Ho: Green)**

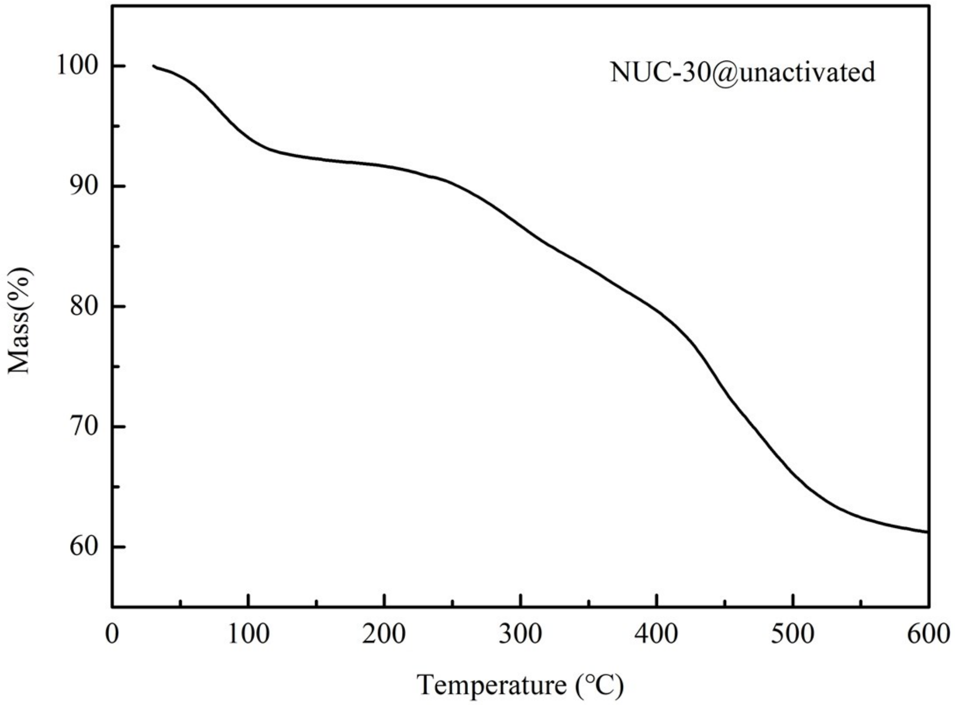


**Figure S4. TGA curve of as-synthesized sample of NUC-30.**

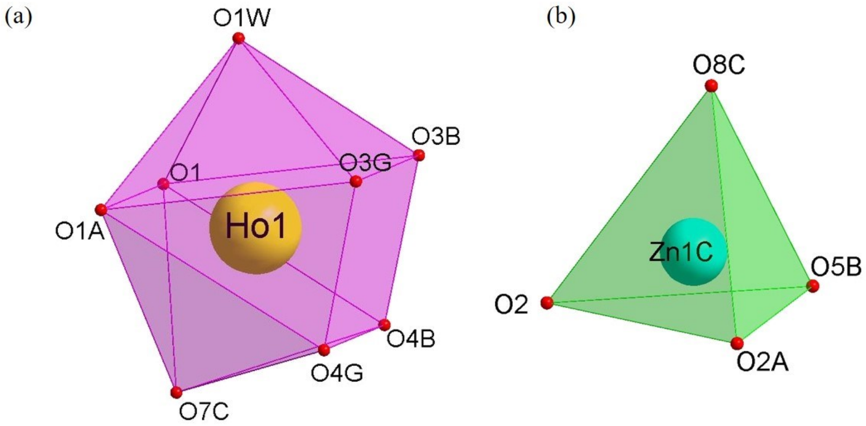


**Figure S5. Ho-o polyhedral structure(a), Zn-o tetrahedral structure(b).**

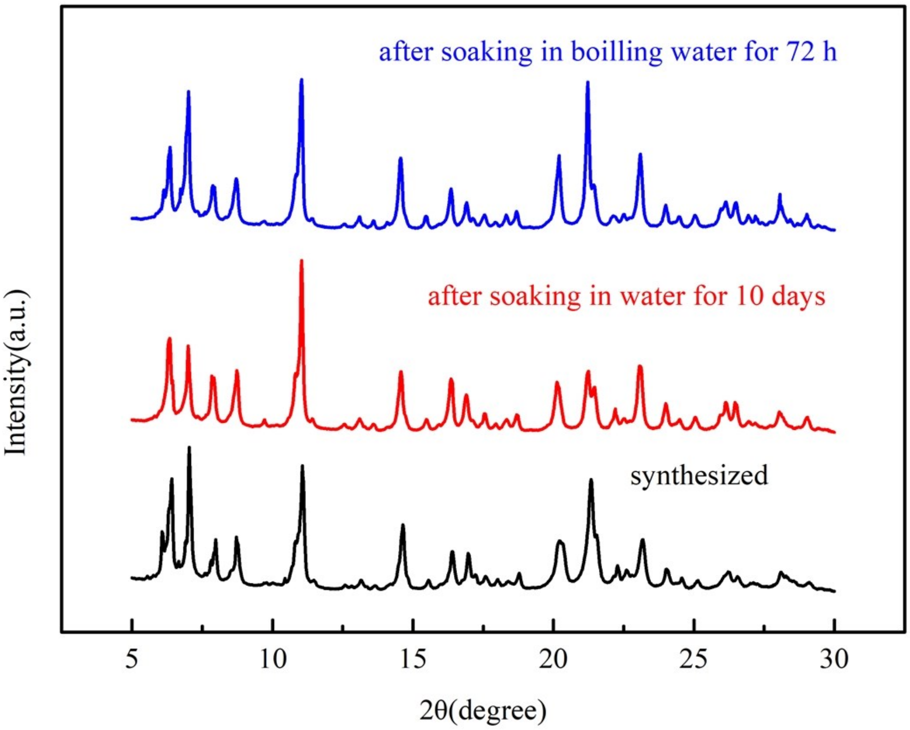


**Figure S6. PXRD patterns of NUC-30 under water treatment.**

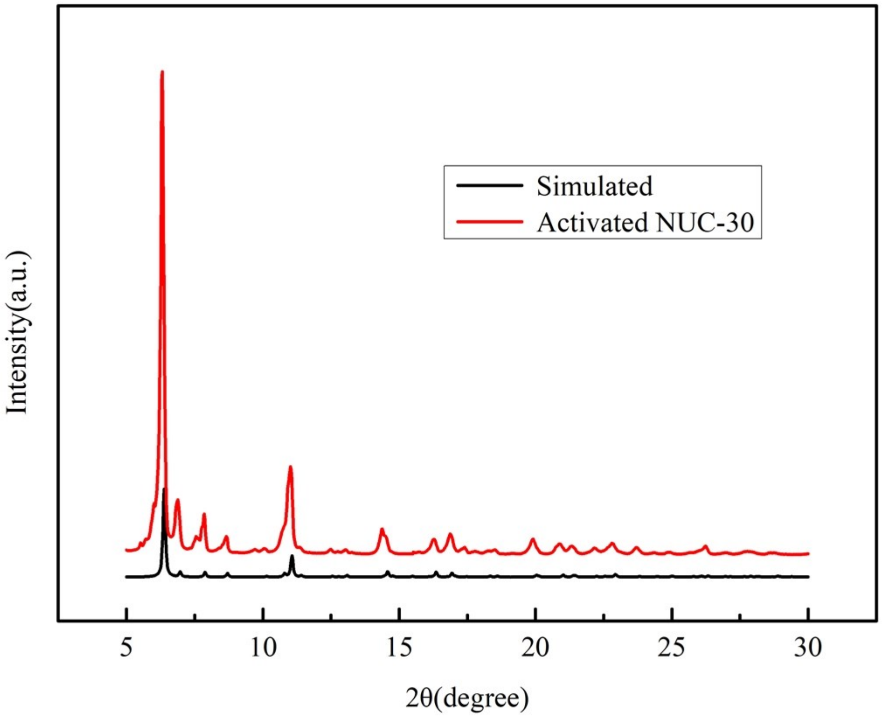

**Figure S7. PXRD pattern of activated NUC-30 and simulated**

**Isosteric Heat Calculation.**

The $Q_{st}$ value is a parameter describing the average adsorption enthalpy of adsorbed gas molecules within a specific surface coverage. It is usually evaluated by two or more adsorption isotherms collected at similar temperatures. The method of calculating the isothermal heat of zero cover adsorption is to first synthesize the temperature related isotherm data into a dimensional expression, which can be written as:

$$lnP = lnN + \frac{1}{T}\sum_{i=0}^{m} a_i N^i + \sum_{i=0}^{n} b_i N^i$$

$$Q_{st} = -R\sum_{i=0}^{m} a_i N^i$$

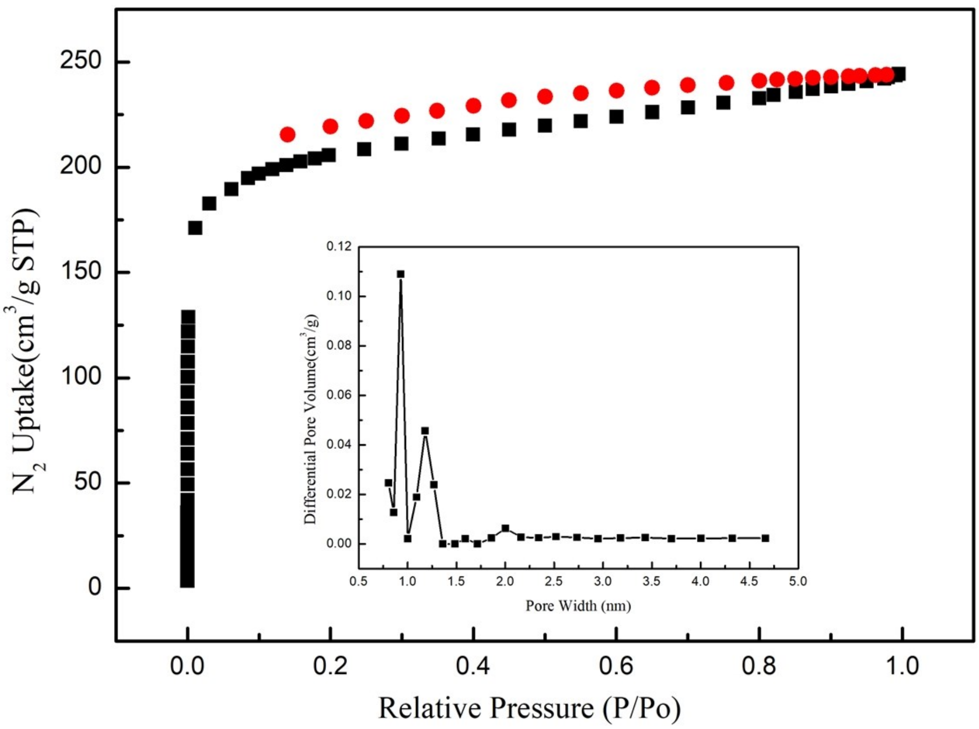


**Figure S8. $N_2$ absorption and desorption isotherms of NUC-30 at 77 K (Insert: the pore size distribution)**

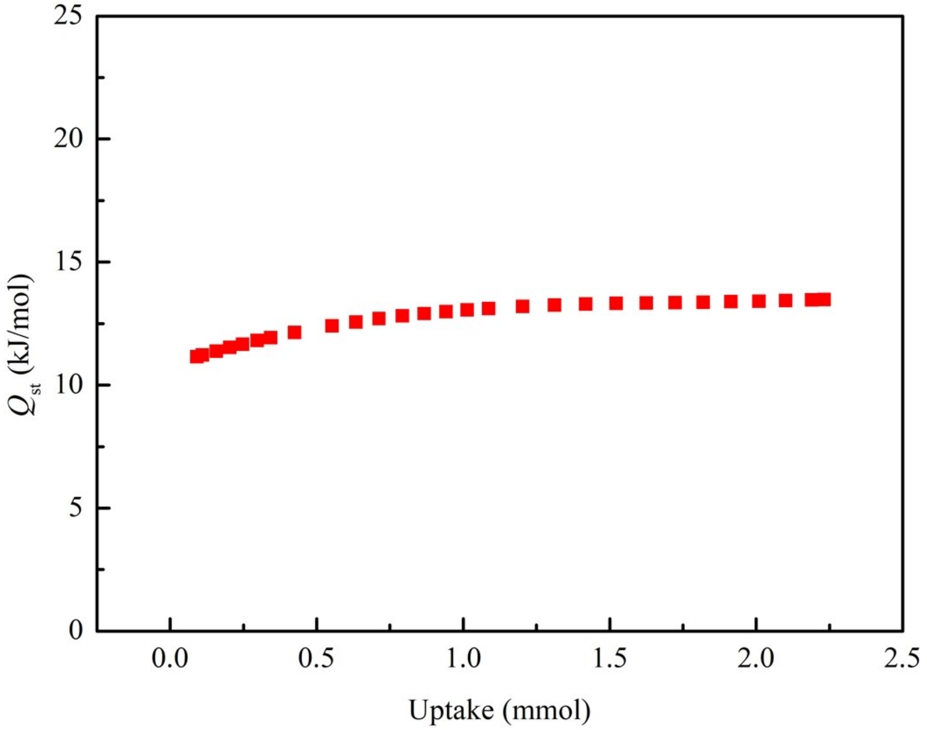


**Figure S9. $CO_2$ adsorption heat calculated by the virial equation of NUC-30.**

**Yield Calculation Based on the GC-MS Analysis**

Gas chromatography mass spectrometry (GC-MS) analyses were executed on a time-of-flight Thermo Fisher Trace ISQ GC/MS instrument, the yield (%) was calculated based on the consumption of starting material using the equation:

$$Yield\ (\%) = \left( \frac{\dfrac{area\ of\ reactant\ at\ 0\ hour}{area\ of\ interal\ standard\ at\ 0\ hour} - \dfrac{area\ of\ reactant\ at\ any\ time}{area\ of\ interal\ standard\ at\ any\ time}}{\dfrac{area\ of\ reactant\ at\ 0\ hour}{area\ of\ interal\ standard\ at\ 0\ hour}} \right) \times 100\%$$

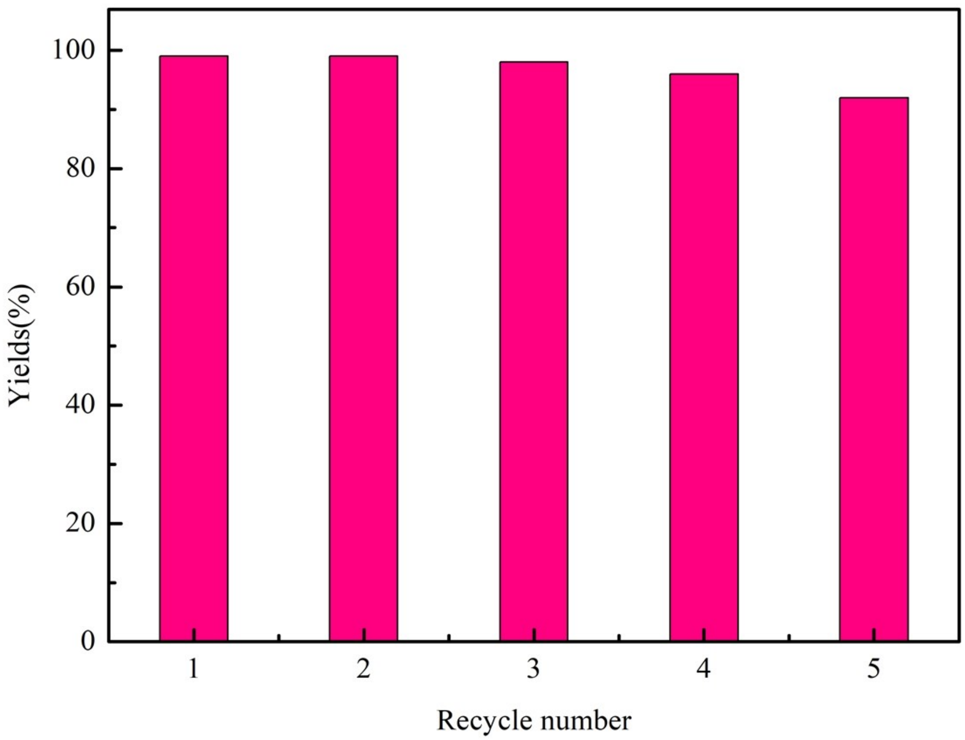


**Figure S10. The recycled cycloaddition reaction of $CO_2$ with styrene oxide.**

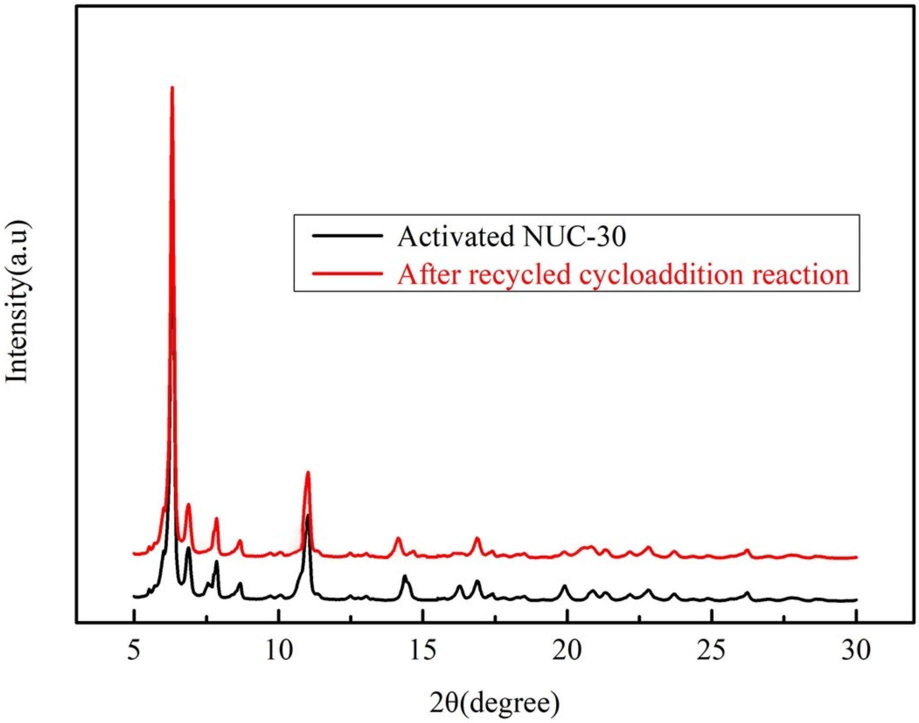


**Figure S11. The PXRD pattern of NUC-30 after recycled cycloaddition reaction.**

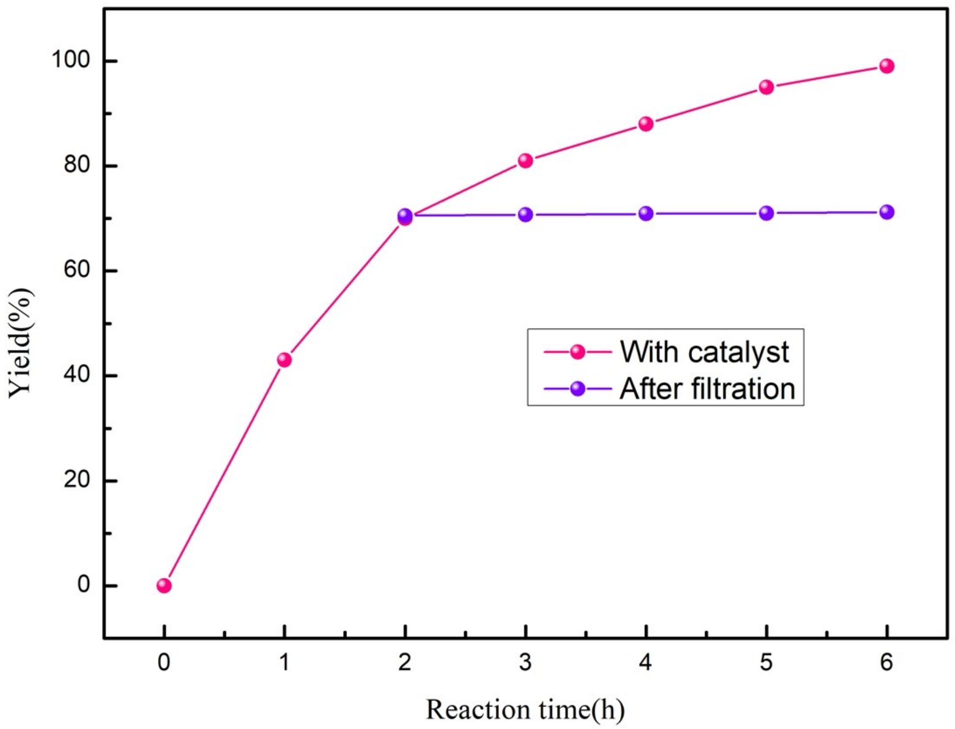


**Figure S12. Evidence of heterogeneous nature of NUC-30 in the coupling of chloromethyloxiran with $CO_2$.**

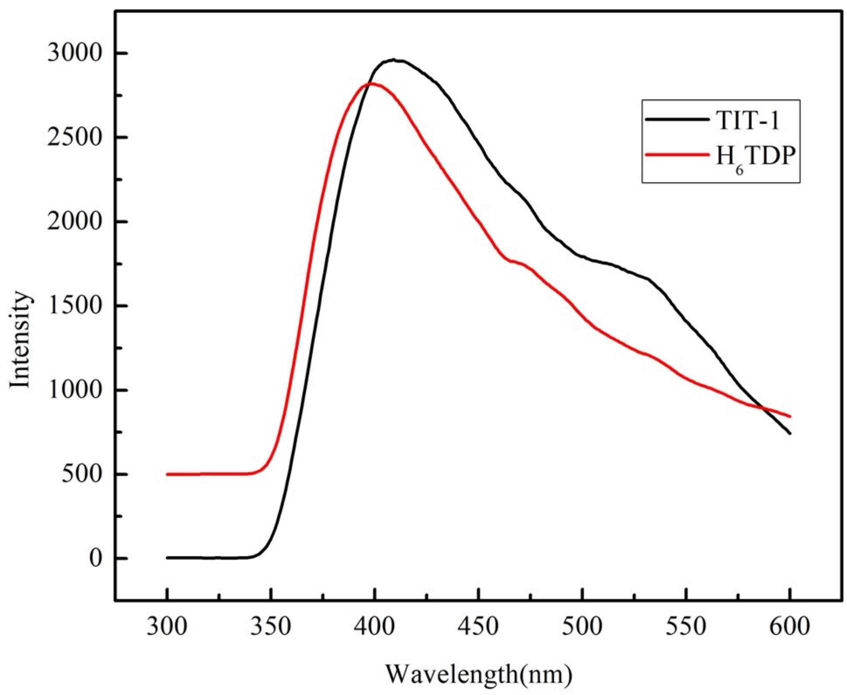


**Figure S13. The fluorescent emission curves of NUC-30(black) and ligand $H_6TDP$ (red).**

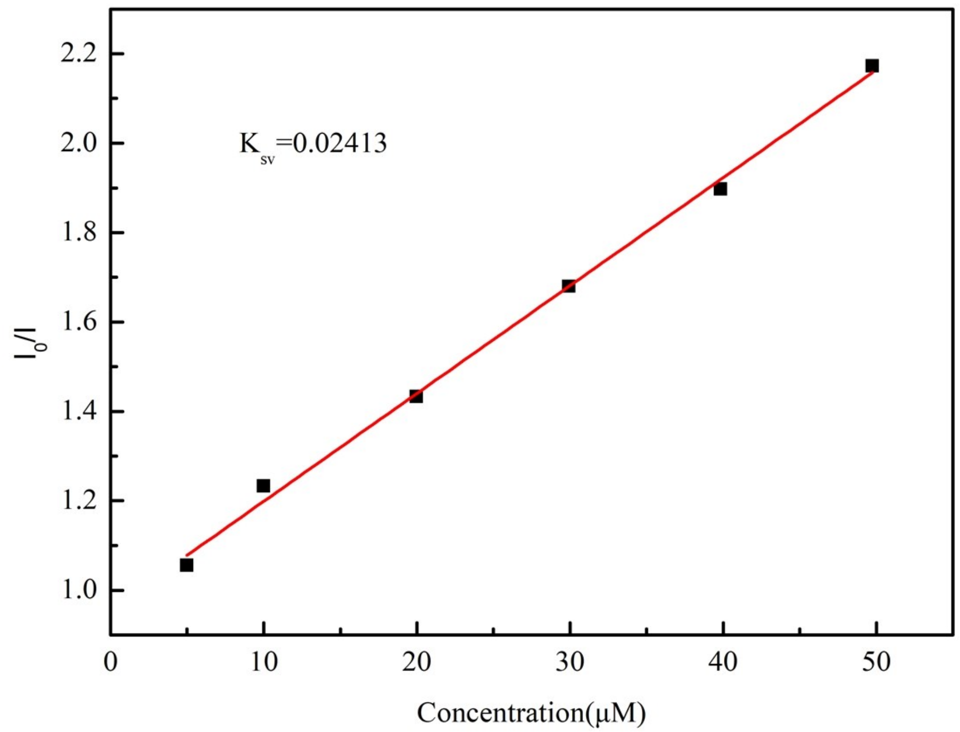


**Figure S14.The fitting experimental data with low concentration range of $Fe^{3+}$.**

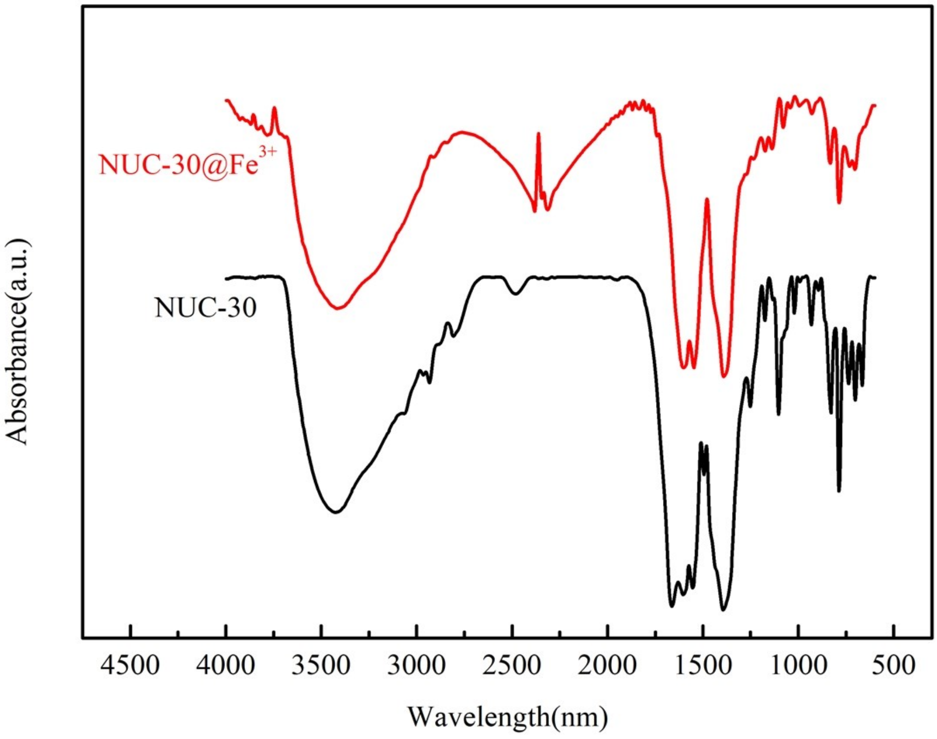


**Figure S15. IR spectrum of NUC-30 under fluorescence quenching of different ions.**

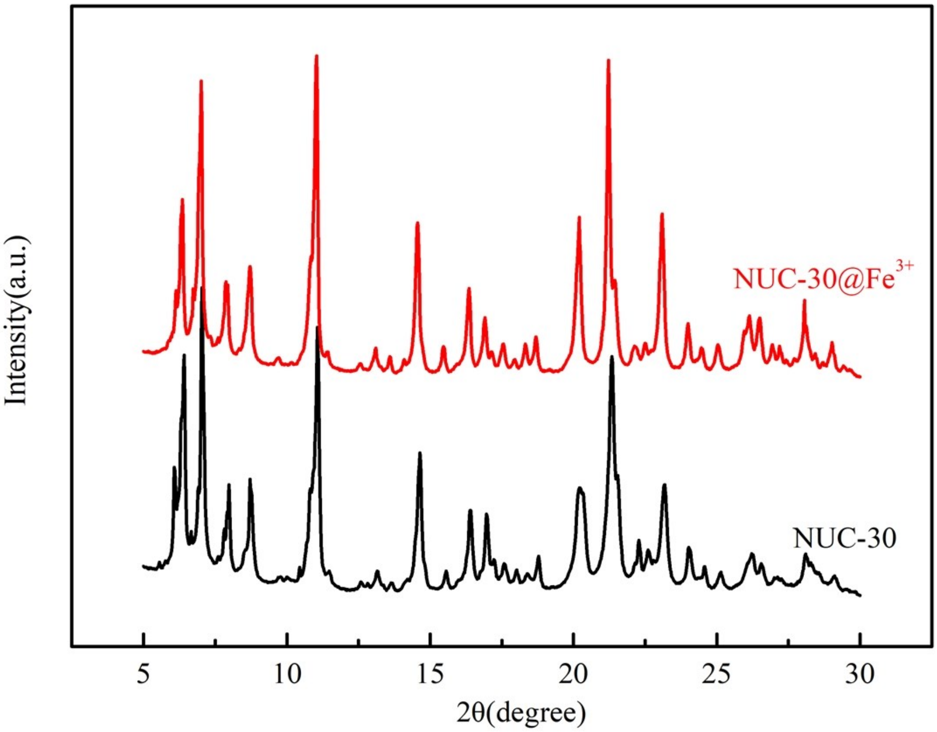

**Figure S16. PXRD patterns of NUC-30 under fluorescence quenching of different ions.**

## Reference


S1. Babu, R. Roshan, R.; Kathalikkattil, A. C.; Kim, D. W.; Park D.-W. Rapid. *ACS Appl. Mater. Interfaces* **2016**, *8*, 33723−33731.

S2. Senthilkumar, S.; Maru, M. S.; Somani, R. S.; Bajaj, H. C.; Neogi, S. *Dalton Trans*. **2018**, *47*, 418−428.

S3. Gao, W. Y.; Chen, Y.; Niu, Y. H.; Williams, K.; Cash, L.; Perez, P. J.; Wojtas, L.; Cai, J. F.; Ma, S. Q. *Angew. Chem., Int. Ed*. **2014**, *53*, 2615−2619.

S4. Zalomaeva, O. V.; Chibiryaev, A. M.; Kovalenko, K. A.; Kholdeeva, O. A.; Balzhinimaev, B. S.; Fedin, V. P. *J. Catal*. **2013**, *298*, 179−185.

S5. Luo-Gang Ding, Bing-Jian Yao, Wei-Ling Jiang, Jiang-Tao Li, Qi-Juan Fu, Yan-An Li, Zhen-Hua Liu, Jian-Ping Ma, Yu-Bin Dong. *Inorg. Chem.* **2017**, *56*, 2337−2344.

S6. Phuong T. K. Nguyen, Huong T. D. Nguyen, Hung N. Nguyen, Christopher A. Trickett, Quang T. Ton, Enrique Gutié rrez-Puebla, M. Angeles Monge, Kyle E. Cordova, Felipe Gá ndara. *ACS Appl. Mater. Interfaces*. **2018**, *10*, 733−744.

S7. Y. B. N. Tran, Phuong T. K. Nguyen, Quang T. Luong, Khoi D. Nguyen. *Inorg. Chem*. **2020**, *59*, 16747−16759.
